# Influence of ultrafiltration conditions on the measurement of unbound drug concentrations: flucloxacillin as an example

**DOI:** 10.1093/jac/dkae092

**Published:** 2024-04-03

**Authors:** Nynke G L Jager, Eleonora Van Ewijk-Beneken Kolmer, Rob Aarnoutse, Lindsey H M Te Brake

**Affiliations:** Department of Pharmacy, Radboud Institute for Medical Innovation, Radboud University Medical Center, Nijmegen, The Netherlands; Department of Pharmacy, Radboud Institute for Medical Innovation, Radboud University Medical Center, Nijmegen, The Netherlands; Department of Pharmacy, Radboud Institute for Medical Innovation, Radboud University Medical Center, Nijmegen, The Netherlands; Department of Pharmacy, Radboud Institute for Medical Innovation, Radboud University Medical Center, Nijmegen, The Netherlands

## Abstract

**Background:**

When performing therapeutic drug monitoring (TDM) for flucloxacillin, it is advised to measure the unbound, not the total, flucloxacillin concentration. To be able to accurately quantify unbound flucloxacillin concentrations, a reliable analytical method is indispensable.

**Objective:**

To determine the influence of temperature and pH of the sample during ultrafiltration on the measured unbound fraction of flucloxacillin.

**Materials and methods:**

We performed three different experiments. In a single laboratory experiment, we investigated the influence of ultrafiltration temperature (10°C, room temperature and 37°C) on the measured unbound fraction of flucloxacillin for three concentration levels. In a multiple laboratory experiment, the results of eight laboratories participating in an international quality control programme measuring unbound flucloxacillin concentrations were analysed. In the third experiment, patient samples were ultrafiltrated using four different conditions: (i) physiological pH and room temperature; (ii) unadjusted pH (pH 9 after freezing) and room temperature; (iii) physiological pH and 37°C and (iv) unadjusted pH and 37°C.

**Results:**

For all experiments, measurement of samples that were ultrafiltrated at room temperature resulted in a substantially lower unbound fraction compared to samples that were ultrafiltrated at 37°C. Adjusting the pH to physiological pH only had a minimal impact on the measured unbound fraction.

**Conclusions:**

On the basis of these findings and considering the need for fast, simple and reproducible sample pretreatment for TDM purposes, we conclude that ultrafiltration of flucloxacillin should be performed at physiological temperature (37°C), but adjustment of pH does not seem to be necessary.

## Introduction

Flucloxacillin is a beta-lactam antibiotic frequently used in the treatment of infections caused by Gram-positive bacteria, such as penicillinase-producing staphylococci. The clinical outcome of treatment with beta-lactam antibiotics is related to the time the unbound (or free) drug concentration remains above the MIC of the targeted pathogen, *f*T > MIC.^1^ Traditionally, total concentrations are measured and unbound concentrations are calculated using protein binding values from the literature. This method is likely to be reliable for drugs with a relatively low protein binding (<80%). However, for flucloxacillin, with a protein binding of ∼90%–95%^2,3^ clinical data have shown that it is not possible to reliably estimate the unbound concentration from the measured total concentration. Therefore, it is advised to measure the unbound flucloxacillin concentration.^4^ When using unbound concentrations for pharmacological research or in clinical practice for therapeutic drug monitoring (TDM) purposes, it is of importance to (i) mimic the *in vivo* bound–unbound equilibrium during the separation of the unbound drug molecules from protein-bound drug molecules, (ii) obtain reproducible results between different laboratories and to (iii) enable a short turnaround time. Different methods are available to obtain unbound drug molecules for quantification, the relatively fast and simple ultrafiltration (UF) method being the most routinely used. To enable adequate measurement of unbound flucloxacillin concentrations for TDM purposes, we investigated the influence of temperature and pH of the sample during ultrafiltration on unbound flucloxacillin quantification.

## Materials and methods

### Bioanalytical assay for the quantification of flucloxacillin

Total and unbound flucloxacillin plasma concentrations were analysed using a UPLC–MS/MS method set up for the measurement of seven commonly prescribed antibiotics, described in a separate report.^5^ In short, for the quantification of total flucloxacillin, sample pretreatment included protein precipitation with a solution containing stable isotopically labelled internal standards including ^13^C_4_-flucloxacillin·Na, in methanol. For the quantification of unbound flucloxacillin, an aliquot of 500 µL of blank plasma, patient sample or quality control (QC) sample was pipetted onto the Centrifree YM-30 ultrafiltration device (Millipore B.V., Amsterdam, the Netherlands), after which it was equilibrated for 60 minutes at 1**g** to reach the aimed sample temperature in the centrifuge (Rotanta 460R, Hettich, Geldermalsen, the Netherlands) when the aimed sample temperature was not room temperature. Consequently, the sample was ultrafiltrated by centrifuging for 20 minutes at 1650**g** according to the instructions of the manufacturer of the UF device. Calibration standards were prepared freshly before each run by spiking blank ultrafiltrate. Sample pretreatment of UF samples included dilution with methanol containing ^13^C_4_-flucloxacillin·Na.

### Influence of temperature during ultrafiltration in spiked samples

#### Single laboratory experiment

QC samples were prepared by spiking blank human plasma with flucloxacillin stock solution of 5 mg/mL in DMSO, resulting in <1% DMSO in plasma, for three concentration levels: low (1 mg/L), mid (5 mg/L) and high (50 mg/L). For five QC samples per concentration level, sample preparation was performed according to the procedure described for total QCs in the section ‘Bioanalytical assay for the quantification of flucloxacillin’. For 15 QC samples per concentration level, sample preparation was performed according to the procedure described for unbound QCs. For five unbound QC samples per concentration level ultrafiltration was performed at 10°C, for five samples per concentration level at room temperature (20°C) and for five QC samples per concentration level at 41°C. In the last case, 41°C was chosen to obtain a sample temperature of 37°C. Consequently, all samples were prepared and analysed as described in the section ‘Bioanalytical assay for the quantification of flucloxacillin’.

#### Multiple laboratory experiment—quality control programme

Twice yearly, the Drug Analysis and Toxicology (KKGT) section of the Dutch Foundation of Quality Assessment in Medical Laboratories (SKML, www.skml.nl) organizes a QC programme that includes measurement of total and unbound flucloxacillin.^6^ In the round of 2023.1, participating laboratories were asked to report the ultrafiltration conditions, i.e. temperature and pH. Sample 2023.1A was spiked with 10.8 mg/L and sample 2023.1B with 50.6 mg/L flucloxacillin.

### Influence of (non-)physiological conditions during ultrafiltration in patient samples

Stored (−40°C) plasma samples previously obtained for TDM purposes from four patients were used. pH was measured in the samples after thawing, showing a pH of ∼9. The patient samples were split into five aliquots, i.e. one sample for measurement of total flucloxacillin and four samples for measurement of unbound flucloxacillin using different UF conditions. For each patient, the pH of two samples was adjusted using a CO_2_ incubator (13% CO_2_) at 37°C (as suggested by^7^), in an open ultrafiltration device, in which an incubation time of 30 minutes was shown to be sufficient to reach pH 7.4. After incubation, the device was immediately closed. Per patient, one pH 7.4 sample and one pH 9 sample were ultrafiltrated at room temperature, and one pH 7.4 sample and one pH 9 sample were ultrafiltrated at physiological temperature. Finally, all samples were prepared and analysed as described in the section ‘Bioanalytical assay for the quantification of flucloxacillin’.

#### Stability of the samples during the experiments

To assess the effect of instability of flucloxacillin during the experiments, total flucloxacillin concentrations were measured in spiked QC samples and patient samples (all prepared in duplicate) after storage at 41°C for 2, 4 and 6 hours.

### Statistical analysis

The unbound fraction was calculated for each individual sample as the ratio of measured concentration in ultrafiltrate to the measured concentration in plasma. Differences between the measured unbound fraction in the different samples was assessed using a non-parametric Mann–Whitney test in the case of two conditions (for the QC programme), and analysis of variance (ANOVA) in the case of more than two conditions (single laboratory experiment and patient samples). Where the ANOVA test was statistically significant, *post hoc* analysis was performed using Tukey’s method to compare all possible group parings.

### Ethics

Ethical approval was obtained from the medical ethics research committee of the Radboudumc (METC East-Netherlands, Nijmegen, the Netherlands) with a waiver for informed consent for the use of anonymous patient samples for this research.

## Results

### Influence of temperature during ultrafiltration in spiked samples

#### Single laboratory experiment

**Figure 1.**
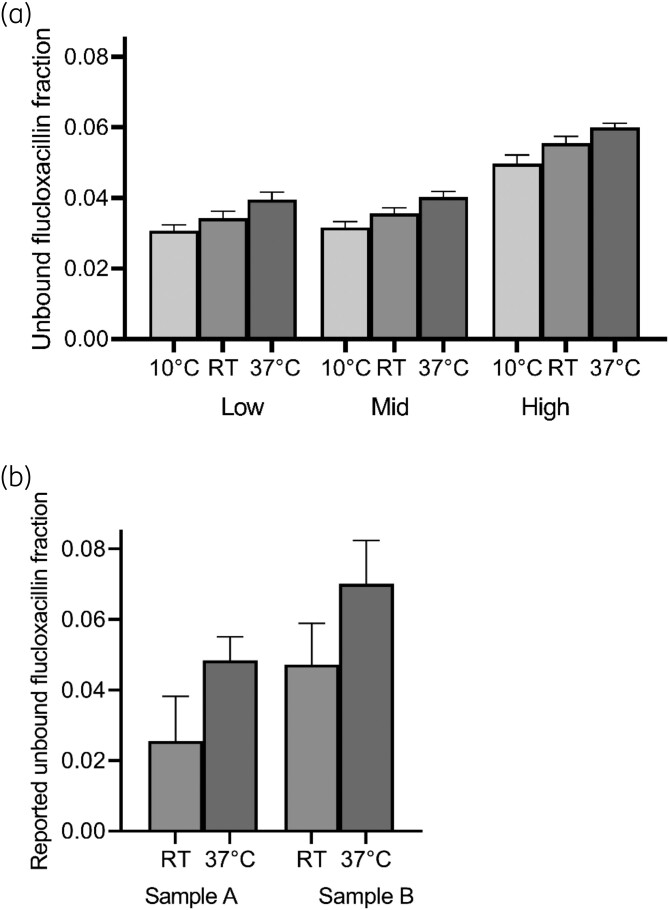
(a) Mean unbound fraction (*n* = 5 for each ultrafiltration temperature and concentration level) measured using three different temperature conditions during ultrafiltration for spiked samples on three total (protein bound + unbound) concentration levels; low 1 mg/L; mid 5 mg/L and high 50 mg/L. (b) Mean unbound fraction measured by different laboratories, performing ultrafiltration at room temperature (RT, *n* = 2 for sample A, *n* = 5 for sample B) or 37°C (*n* = 3) on spiked samples from an international QC programme. Results are shown as mean ± standard deviation.

There was a statistically significant (*P* < 0.05) difference between the unbound fractions of all tested ultrafiltration temperatures for all three concentration levels. In all samples, the measured unbound concentration increased with higher temperatures during UF. Results are shown in Figure 1(a).

#### Multiple laboratory experiment—quality control programme

Eight participating laboratories reported the used UF conditions. Five laboratories reported performing UF at room temperature and three laboratories reported performing UF at 37°C. None of the laboratories reported that they adjusted the pH of the plasma sample before ultrafiltration. For sample A, three out of five laboratories that reported performing UF at room temperature reported an unbound concentration below the lower limit of quantification of their assay, and were excluded from the statistical analysis for this sample. Of note, two of these three laboratories reported a lower limit of quantification below the mean measured unbound concentration of the three laboratories performing UF at 37°C. Results are depicted in Figure 1(b); the mean reported unbound fraction was 49% lower for sample A ultrafiltrated at room temperature (*n* = 2, range 0.017–0.034) compared to UF at physiological temperature (*n* = 3, range 0.043–0.056) and 35% lower for sample B ultrafiltrated at room temperature (*n* = 5, range 0.033–0.061) compared to UF at physiological temperature (*n* = 3, range 0.058–0.082). Owing to the low sample size and relatively high variability between different laboratories, these differences were not statistically significant for both samples A (*P* = 0.2) and B (*P* = 0.07).

### Influence of (non-)physiological conditions during ultrafiltration in patient samples

**Figure 2.**
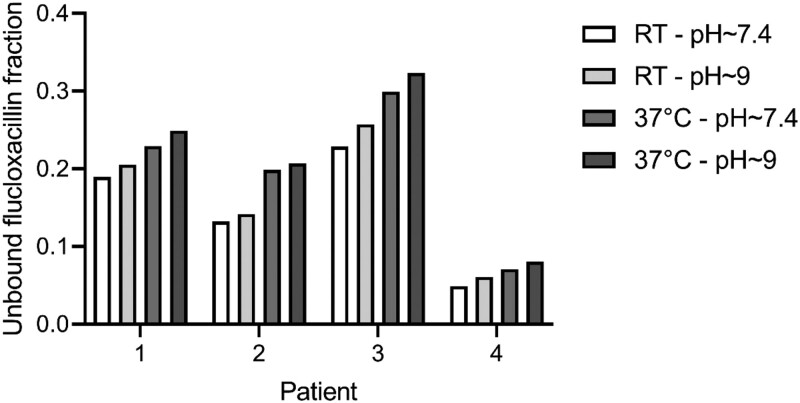
Unbound fraction measured in TDM samples obtained from four patients. Ultrafiltration was performed using four different conditions for each sample; (i) room temperature (RT) and adjusted pH to 7.4; (ii) room temperature and unadjusted pH (pH 9); (iii) physiological temperature (37°C) and adjusted pH to 7.4 and (iv) physiological temperature (37°C) and unadjusted pH (pH 9).

Measured total flucloxacillin concentrations were 71.0 mg/L for patient 1, 78.4 mg/L for patient 2, 41.4 mg/L for patient 3 and 49.2 mg/L for patient 4. The unbound fractions in the samples that were ultrafiltrated at room temperature were 18%–32% lower than in samples that were ultrafiltrated at physiological temperature. The unbound fractions in the samples that were ultrafiltrated without adjustment of the pH (i.e. pH ∼ 9) were 4%–13% higher than in samples with pH 7.4. See Figure 2 for the results. The observed differences were not statistically different between the different UF conditions when the results of the four patients were combined.

#### Stability of the samples during the experiments

For QC samples, the mean measured flucloxacillin concentration was 96% (range 88%–105%) after 2 hours, 83% (79%–87%) after 4 hours and 66% (59%–71%) after 6 hours at 41°C.

For patient samples, the mean measured flucloxacillin concentration was 92% (range 88%–93%) after 2 hours, 81% (76%–85%) after 4 hours and 62% (54%–70%) after 6 hours.

## Discussion and conclusions

This research shows the importance of investigating different UF conditions for measuring unbound drug concentrations. Our findings are in line with several other reports, all concluding that temperature during UF influences the measured unbound antibiotic concentration.^8–10^ In two of these studies, flucloxacillin was investigated, and similar results to our findings were obtained; in the first study, a significantly lower unbound concentration (27%) was reported in spiked samples that were ultrafiltrated at room temperature versus UF at 37°C.^8^ The second study showed a 13%–15% lower unbound fraction at room temperature compared to 37°C in pooled patient samples.^9^

Our results indicate a minimal impact of pH on the measurement of unbound flucloxacillin. The slightly lower unbound concentration in samples ultrafiltrated at pH 7.4 versus at pH 9 may be partly explained by instability of flucloxacillin at 37°C, considering the samples were incubated for 30 minutes in the CO_2_ incubator at 37°C to lower the pH to ∼7.4.

This is the first report where the influence of UF conditions in both spiked samples and patient samples was investigated, as well as analyses performed in different laboratories. On the basis of these findings and considering the need for fast and simple sample pretreatment for TDM purposes, we conclude that UF of flucloxacillin should be performed at physiological temperature (37°C), but adjustment of pH seems to be unnecessary. This research can be used as an example for other drugs for which unbound concentrations are used in clinical practice. We emphasize the need for investigation of the UF conditions to ensure the physiological bound–unbound equilibrium is maintained, before implementing the measurement of unbound drug concentrations in clinical practice.
